# Polymer-Functionalised Nanograins of Mg-Doped Amorphous Calcium Carbonate via a Flow-Chemistry Approach

**DOI:** 10.3390/ma12111818

**Published:** 2019-06-04

**Authors:** Benedikt Demmert, Frank Schinzel, Martina Schüßler, Mihail Mondeshki, Joachim Kaschta, Dirk W. Schubert, Dorrit E. Jacob, Stephan E. Wolf

**Affiliations:** 1 Department of Materials Science and Engineering (WW), Institute of Glass and Ceramics (WW3), Friedrich-Alexander University Erlangen-Nuremberg (FAU), Martensstrasse 5, D-91058 Erlangen, Germany; benedikt.bd.demmert@fau.de (B.D.); frank.schinzel@googlemail.com (F.S.); martina.schuessler@fau.de (M.S.); 2 Department of Earth and Planetary Sciences, Macquarie University, Sydney, NSW 2109, Australia; dorrit.jacob@mq.edu.au; 3 Institute for Inorganic and Analytical Chemistry, Johannes Gutenberg-University, Duesbergweg 10-14, 55128 Mainz, Germany; mondeshk@uni-mainz.de; 4 Department of Materials Science and Engineering (WW), Institute of Polymer Materials (WW5), Friedrich-Alexander University Erlangen-Nuremberg (FAU), Martensstrasse 5, D-91058 Erlangen, Germany; joachim.kaschta@fau.de (J.K.); dirk.schubert@fau.de (D.W.S.); 5 Interdisciplinary Center for Functional Particle Systems (FPS), Friedrich-Alexander University Erlangen-Nuremberg, 91058 Erlangen, Germany

**Keywords:** amorphous calcium carbonate, flow-chemistry, nanoceramics, biomaterials, microfluidics

## Abstract

Calcareous biominerals typically feature a hybrid nanogranular structure consisting of calcium carbonate nanograins coated with organic matrices. This nanogranular organisation has a beneficial effect on the functionality of these bioceramics. In this feasibility study, we successfully employed a flow-chemistry approach to precipitate Mg-doped amorphous calcium carbonate particles functionalized by negatively charged polyelectrolytes—either polyacrylates (PAA) or polystyrene sulfonate (PSS). We demonstrate that the rate of Mg incorporation and, thus, the ratio of the Mg dopant to calcium in the precipitated amorphous calcium carbonate (ACC), is flow rate dependent. In the case of the PAA-functionalized Mg-doped ACC, we further observed a weak flow rate dependence concerning the hydration state of the precipitate, which we attribute to incorporated PAA acting as a water sorbent; a behaviour which is not present in experiments with PSS and without a polymer. Thus, polymer-dependent phenomena can affect flow-chemistry approaches, that is, in syntheses of functionally graded materials by layer-deposition processes.

## 1. Introduction

Calcium carbonate is of fundamental importance in various fields of research, even beyond its industrial and biological abundance [1,2]. For instance, it is of key relevance as a proxy archive in reconstructing past climates or serves as a cement additive or for exploration of biomimetic mineralisation processes [3,4,5,6,7,8,9,10]. Increasingly, calcium carbonate is suggested and introduced as an alternative to calcium phosphate-based biomaterials: Its application as a bone replacement material [11,12,13,14] and as a drug delivery system is currently explored [15,16,17,18,19,20], and it plays a central role for the bioactivity of bioglass [21]. Due to its central relevance in these various fields, the underlying mechanisms of its formation consequentially attracted broad scientific interest. As of yet, its formation mechanism is still in debate [22,23,24,25]; the binary system calcium carbonate/water is exceptionally prone to so-called nonclassical crystallization processes, namely to mineralization routes which are driven by accretion of larger entities (e.g., nanoparticles) [26], instead of single ions as predicted by classical theories. Amorphous calcium carbonate (ACC) is commonly observed in such nonclassical crystallisation pathways [27,28,29], and it is a remarkable coincidence that biomineralizing organisms, which form their functional bioceramics from calcium carbonate, typically employ ACC as an initial building material [28,30,31]. ACC allows for the generation of non-equilibrium morphologies by pseudomorphic transformation and the incorporation of dopants and organic matrices into the final biomineral [28,32,33,34]. In several biominerals, remnants of amorphous calcium carbonate are found even in mature specimens [28,30,35,36,37].

We recently gave first direct evidence for a nonclassical and thus nanoparticle-driven mineralisation pathway in a biomineralisation system [38]. For the prominent case of nacre, we revealed by scanning transmission electron microscopy analysis that the self-organisation of calcium carbonate colloids drives nacre growth in the model system of the Mediterranean bivalve *Pinna nobilis*. The typical size of these colloids lines up perfectly with the characteristic size of nanograins, which are the fundamental building units of biominerals of a large number of diverse species [23,35,36,39]. These nanograins are coated by an organic matrix which effectively turns the biomineral into a hybrid material. Moreover, the nanogranular organisation turns the biomineral into a nanoceramic, which has a range of beneficial effects on the biomineral’s properties, of which the mechanical aspects are especially well documented [23]. According to Griffith’s law, a brittle material can reach its theoretical strength by reducing the grain size below a critical threshold in the nanometer range [40,41,42]. Additionally, the nanogranular structure leads a crack onto a tortuous intergranular trail that distinctly increases the crack path [43,44]. Since the intergranular organic matrix enriches at the nanograin boundaries, it can efficiently increase the crack energy dissipation considerably, for example, by ligament bridging [44].

In summary, biominerals often feature a nanogranular organisation which arises from nanoparticle-driven mineralisation, and hence nonclassical pathway in which amorphous calcium carbonate colloids accrete to give a space-filling and, after pseudomorphic phase transformation, crystalline mineral body; the colloidal origin of these functional bioceramics imprints a nanogranular structure on these materials [23,45]. At the nanograin boundaries, organic matrices are incorporated, affecting the bioceramic properties fundamentally. Ultimately, the intracrystalline and intergranular organic matrices transform the calcium carbonate body into a hybrid nanoceramic.

The mimesis of the biosynthesis of such hybrid materials often builds upon diffusion-controlled single-batch mineralisation setups in which, following the concept of LaMer [46], a high supersaturation generates a high particle number density which then self-organises into nanostructured mineral bodies and mesocrystals [30,32,47,48,49]. These systems often yield materials which are remarkably similar to their biogenic counterparts [49], but the chosen synthesis concept also has various major drawbacks. The one-batch synthesis approach defines only the initial reaction parameters but, in the course of the reaction, key parameters like pH or supersaturation or ionic strength may change in an unsupervised fashion. Even established systems such as the slow-diffusion process, in which a calcium-bearing solution is exposed to the vapour of decomposing ammonium carbonate, bear hidden parameters which affect reproducibility across different labs [50]. This lack of control not only impedes upscaling but also hinders an intentional change in reaction parameters during the reaction. The latter would allow designing mineralisation procedures which give access, *inter alia*, to graded functional materials. This is already excellently demonstrated by nature’s functional graded biominerals, which are highly adapted to serve a given task [51,52].

In general, chemical reactions such as precipitation reactions by metathesis are governed by concentration profiles which fundamentally alter when changing the experimental design from a batch-wise to a flow-through setup. To overcome the established but limited single-batch setups in biomimetic mineralization experiments and to gain more parametric control over precipitation reactions, we explored the feasibility of a flow-chemistry approach herein. By relying on commercially available microfluidics channels, we ensured that the approach can also be adopted when no access to lithographical technology is available. As a benchmarking model system, we chose a relatively delicate synthesis task, the generation of amorphous calcium carbonate which is subsequently surface-functionalised with representative polyelectrolytes, here either polyacrylate (PAA) or polystyrene sulfonate (PSS), without inducing phase transformation of the highly reactive amorphous phase. Thus, we aimed at generating polymer-functionalized nanograins of Mg-doped amorphous calcium carbonate particles, mimicking the fundamental building blocks of biogenic nanogranular functional ceramics to demonstrate the reliability of a flow-chemistry approach for biomimetic crystallization and bio-inspired materials synthesis.

## 2. Materials and Methods

**Figure 1:**
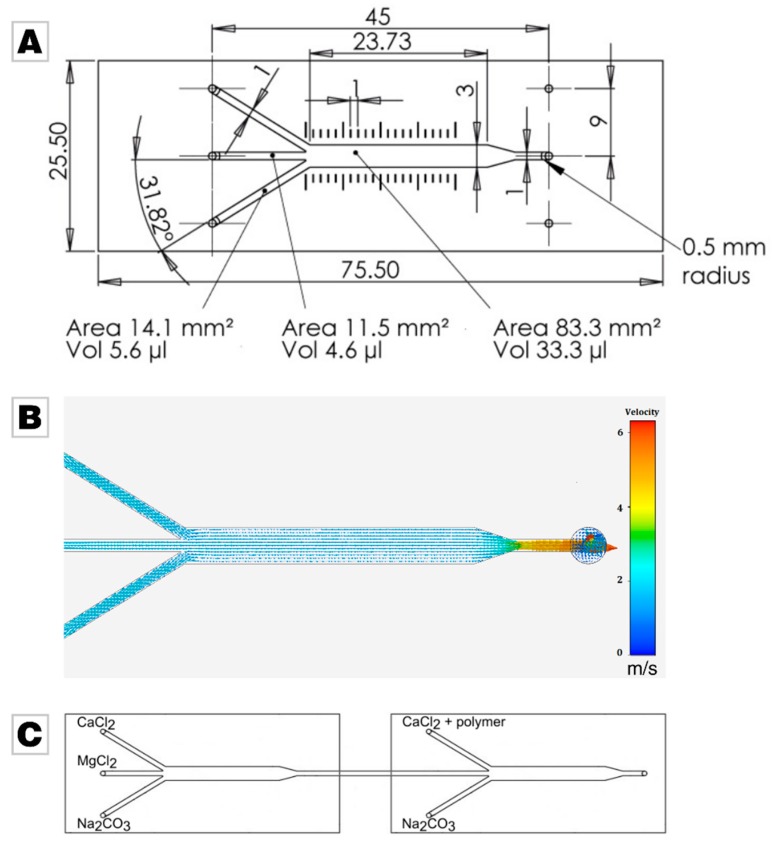
(**A**) Layout of the commercially available and employed microfluidic chip supplied by ibidi (Martinsried, Germany). Image used by courtesy of ibidi GmbH [53]. (**B**) Simulation of the fluid dynamics in the microfluidic chip at a feeding flow of 30 mL/min. Images used courtesy of ANSYS, Inc. (Canonsburg, PA, USA) (**C**) Scheme of the experimental setup. In control experiments, only the calcium chloride solution was fed into the system in the second microfluidic chip, thus omitting the addition of polymer.

The experimental setup consisted of two commercially microfluidic channels which merged three separately fed liquids into a single channel (ibidi GmbH, µ-Slide III3in1); the layout is given in Figure 1A. Simulation of fluid dynamics in the microfluidic chip at a flow rate of 30 mL/min shows that the middle part of the chip features a laminar flow profile, and the orifice induces a turbulent flow with thorough mixing (Figure 1B). For the simulation, the software ANSYS AIM, Release 19.0, was used [54]. The two microfluidic elements were connected in series by standard tubing, see Figure 1C. To control the different flow rates (5 mL/min, 15 mL/min and 30 mL/min) and the volume (20 mL), the solutions were mixed with a peristaltic pump (MA1 70-7000R, Harvard Apparatus, Holliston, MA, USA; tubing size 2.79 mm).

Ultrapure water was used in all experiments (Milli-Q Direct 8 with UV photooxidation, Merck Millipore, Burlington, MA, USA 18.2 MΩ cm^−1^); all other reagents were used as supplied without further purification (purity >99%; Sigma-Aldrich, St. Louis, MO, USA). In the first microfluidic element, Mg-doped ACC was precipitated by mixing a 40 mM CaCl_2_ solution simultaneously with a 0.1 M MgCl_2_ solution and a 40 mM Na_2_CO_3_ solution. In the second microfluidic chip, a 40 mM Na_2_CO_3_ solution and a 40 mM CaCl_2_ solution were added; the CaCl_2_ solution contained additionally 200 µg/mL sodium polyacrylate (henceforth abbreviated as PAA, Sigma Aldrich, M_w_ ~ 5100, pK_A_ ≈ 4.5 [55]) or 82.4 µg/mL, sodium poly(4-styrenesulfonate) (henceforth abbreviated as PSS, Sigma Aldrich, M_w_ ~ 70,000, pK_A_ ≈ 1.0 [56]). As a control experiment, no polymer was added in the second microfluidic chip. After mixing, the solutions were immediately filtered using nitrocellulose membranes (GVS North America), and the received precipitate was rinsed with ethanol and stored in a desiccator over freshly dried silica gel.

Phase analysis was accomplished by X-ray diffraction (D8 Advance Eco, Bruker Corporation, Billerica, MA, USA; Cu Kα source). Measurements were conducted in the 2θ range between 20° and 70° with a step size of 0.05° and a dwell time of 0.3 s. The sample morphologies were analysed by scanning electron microscopy (SEM, GeminiSEM 500, Carl Zeiss, Oberkochen, Germany). The gold sputtered samples were analysed at an accelerating voltage of 1.0 kV and a working distance of 6.5 mm; the final micrographs were evaluated with ImageJ2 [57]. The incorporation of magnesium into amorphous calcium carbonate evidenced by inductively coupled plasma optical emission spectroscopy (ICP-OES, Genesis FES, Spectro Analytical Instruments, Kleve, Germany). For each ICP-OES measurement, 30 mg of powder was dissolved in nitric acid (1 mol L^1^). Every sample was measured at least in triplicate. The polymer incorporation was probed by attenuated total reflection Fourier transform infrared (ATR-FTIR, Nicolet IS10, Thermo Scientific, Waltham, MA, USA accumulation of 64 scans) and thermogravimetry (TGA, TGA Q5000, TA Instruments, New Castle, DE, USA, 5 K/min under nitrogen atmosphere). Before TGA measurements were made, the samples were dried for 24 h at 60 °C to inhibit the influence of water on the measurements.

Furthermore, magic-angle-spinning cross-polarisation solid-state nuclear magnetic resonance (CP-MAS-NMR) experiments were performed (Advance DSX 400, Bruker Cooperation, Billerica, MA, USA, 10 kHz spinning, 3 s recycle delay, 2 ms CP pulse and between 24 and 26 k scans). The low amount of PAA and PSS and the 1% ^13^C natural abundance hardly facilitated a successful recording of ^13^C single pulse (SP) excitation spectra. SP experiments, although quantitative, are related to (sometimes extremely) long recycle delays; for crystalline carbonates containing water, they typically exceed 150 s [58]. In the case of water-free crystalline carbonates, recycle delays easily exceed 1000 s [59]. Thus, quantitative information about the content of PAA in the measured samples is not accessible. For this reason, ^13^C cross-polarisation (CP) NMR spectra of the PAA and PSS samples were recorded at 10 kHz magic angle spinning (MAS) frequency and under heteronuclear decoupling, averaging 20 k scans. Such experiments are routinely measured at spinning speeds of 20 kHz or even higher [60,61]. However, moderate spinning speeds in the range of 8–12 kHz, at which the dipolar interaction is not significantly affected, facilitate optimum cross-polarisation and are still sufficient to average chemical shift anisotropy (CSA). This results in less or no spinning sidebands. Moreover, the ^1^H-^13^C CP experiments are related to the much faster ^1^H relaxation, which extremely reduces the experimental time to provide sufficient but qualitative information about the species present in the system under investigation.

## 3. Results and Discussion

In this feasibility study, we aimed at generating Mg-stabilized ACC nanoparticles (see Appendix A) and subsequently coating them with a second layer of ACC containing a polyelectrolyte in order to mimic the fundamental building blocks of hybrid nanogranular biominerals. A flow-chemistry setup was chosen, which segmented the synthesis into two consecutive steps—nanoparticle synthesis and nanoparticle coating. For this, we chose commercially available microfluidic channels, which feature a laminar flow section, which ends in a smaller orifice causing thorough mixing (see Figure 1). Each of the subsequent reaction steps was accomplished in one of the two consecutive microfluidic channels, which were connected in series. In the second step of nanoparticle coating, we employed two representative negatively charged polyelectrolytes, that is, poly (styrene sulfonate) (PSS) and poly (acrylate) (PAA). In the following, we demonstrate that the chosen approach gives Mg-doped ACC particles in which the polymer can be readily traced.

### 3.1. Powder Characterisation and Validation of Mg Incorporation in Polymer-Functionalized Mg-Doped ACC

**Figure 2:**
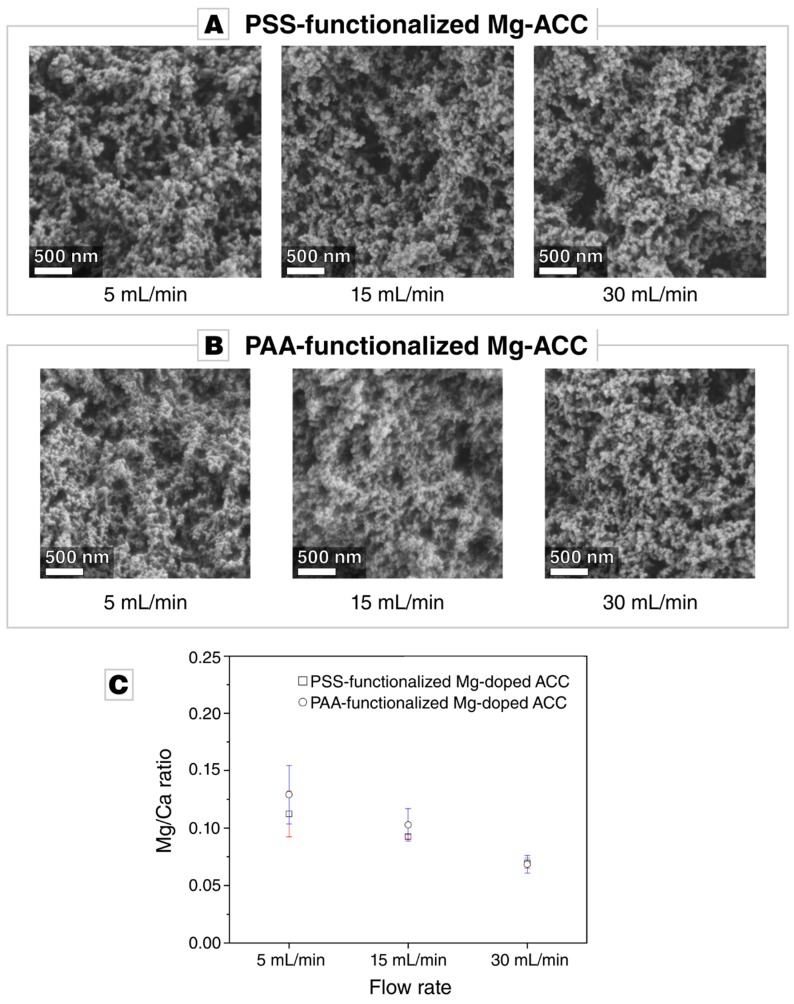
(**A**,**B**) Micrographs acquired by scanning electron microscopy of Mg-doped amorphous calium carbonate (ACC) functionalized by different polymers, i.e., sodium poly(4-styrenesulfonate) (PSS) or sodium polyacrylate (PAA), synthesized at different flow rates: 5 mL/min, 15 mL/min, and 30 mL/min. The morphology of the precipitate is unaffected by the flow rate and the polymer additive. (**C**) Inductively coupled plasma optical emission spectroscopy (ICP-OES) measurements of the doped ACC samples generated at different flow rates. The rate of Mg uptake is unaffected by the chemism of the polymer used for functionalization. With an increasing flow rate, the uptake of magnesium into the amorphous precipitate decreases.

The precipitated Mg-doped calcium carbonate is amorphous, irrespective of the flow rate and the nature of the polymeric additive (Figure S1 in the Supplementary Materials). The particle size of the precipitated ACC is in the range of 100 to 200 nm (Figure 2A,B), which is the typical particle size range of ACC and congruent to the nanograin size in biominerals [23,45,62]. The morphology of the precipitate is influenced neither by the flow rate nor by the added polyelectrolyte (see Figure 2A,B). Magnesium was incorporated in the amorphous calcium carbonate powders at a relatively low Mg/Ca ratio of around 0.1, despite the high MgCl_2_ concentration in the mother solution (see Figure 2C). The flow rate distinctly affects the rate of Mg incorporation: the Mg/Ca ratio decreases with increasing flow rate. The Mg/Ca remains approximately constant when replacing PAA for PSS (Figure 2C).

### 3.2. Validation of PSS Incorporation in Mg-Doped ACC

**Figure 3:**
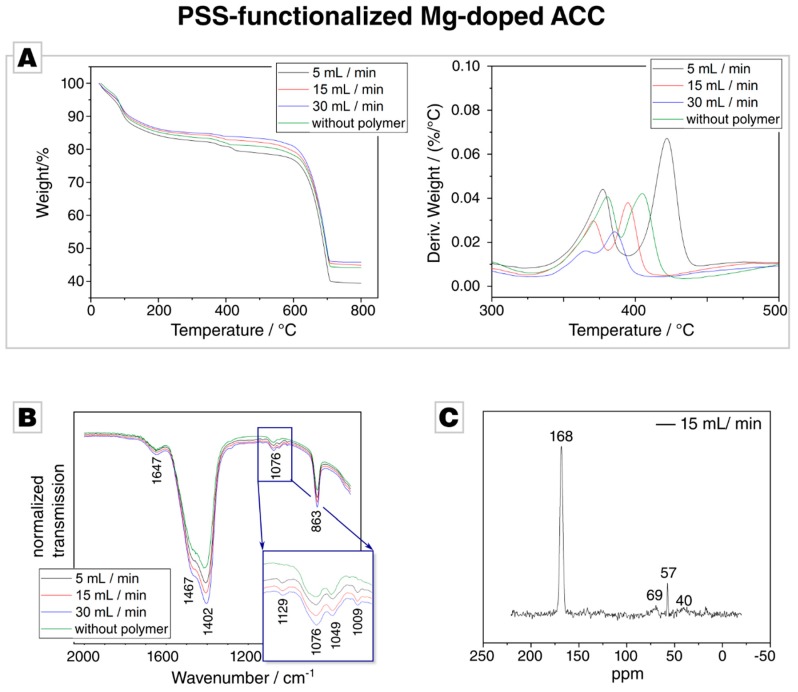
(**A**) Thermal gravimetric analysis (TGA) of ACC precipitated in the presence of PSS at different flow rates. (**B**) Attenuated total reflection Fourier transform infrared (ATR-FTIR) of the PSS and the Mg-doped control sample; the complete spectrum is provided in Figure S3A in the Supplementary Materials. (**C**) Cross-polarisation solid-state nuclear magnetic resonance (CP-MAS ^13^C–SS-NMR) of PAA-functionalized Mg-doped ACC synthesized at 15 mL/min. Other signals, e.g., at 57 ppm, arise from trace impurities such as residual ethanol or are insignificant to the signal/noise ratio.

TGA showed a distinct weight loss of 10% is due to loss of weakly bound water (Figure 3A) when analysing ACC, which was precipitated in the presence of PSS. A detailed derivative analysis revealed that with an increasing flow rate, the hydration rate of the PSS samples decreases. This is consistent with the results from ICP-OES, which showed a decreasing incorporation of magnesium with higher flow rates. At 600 °C, thermal decomposition of CaCO_3_ into CaO and CO_2_ occurs, which results in a sharp weight loss. Between 200 °C and 600 °C, a gradual weight loss is visible, which we attribute to the presence of PSS in the samples. The weight loss is dependent on the flow rate, the lower the flow rate, the higher the weight loss in the given temperature window. A distinct kink is present around 400 °C, which one could attribute to the thermolysis of PSS, since PSS degradation occurs between 410 °C and 470 °C [63]. However, the kink is also present in the control reaction, in which PSS-free Mg-doped ACC was decomposed, which indicates that this weight loss does not originate from an organic component. The derivate of the weight loss shows a double peak, and the onset of the first signal is at 350 °C which coincides well with the thermal decomposition temperature of MgCO_3_ [64]; the weight loss at around 400 °C decreases with increasing flow rate, which lines up well with the decrease in Mg/Ca ratio with increasing flow rate. We, therefore, attribute the presence of this kink to the presence of Mg in the precipitate, which further corroborates our findings on the inverse dependence of Mg incorporation on the flow rate. Overall, these results demonstrate the presence of Mg in the precipitate, but the incorporation of PSS could not be established by TGA.

ATR-FTIR measurements were able to reveal the presence of PSS in the Mg-doped ACC. The control samples and the PSS-functionalized ACC show the expected vibration band of ACC (Figure 3C) [62,65,66,67]. In the region between 1200 cm^−1^ and 900 cm^−1^, the PSS-functionalized ACC samples show two additional bands which are absent in the control sample (inset in Figure 3C), at 1129 cm^−1^ and 1009 cm^−1^. Both bands arise from the C-H bending vibration within the benzene rings [68] and are also present in ATR-FTIR spectra of the pure PSS polymer (Figure S2A in the Supplementary Materials). This eventually gives evidence of the presence of PSS in the Mg-doped ACC.

CP-MAS ^13^C–SS-NMR on samples precipitated at 5, and 15 mL/min was conducted in order to provide evidence for PSS incorporation (Figure 3C). However, the resonances of backbone CH and CH_2_ groups at ca. 41 and 47 ppm and of the aromatic carbons at ca. 128 and 146 ppm were absent, probably due to the very low fraction of incorporated polymer [69]. Besides resonances arising from residual ethanol at 57 ppm [70], only one strong resonance related to carbonate is observed at 168.3 ppm; it features a constant width at half height of ca. 400 Hz across all samples [32,71,72].

### 3.3. Validation of PAA Incorporation in Mg-Doped ACC 

**Figure 4:**
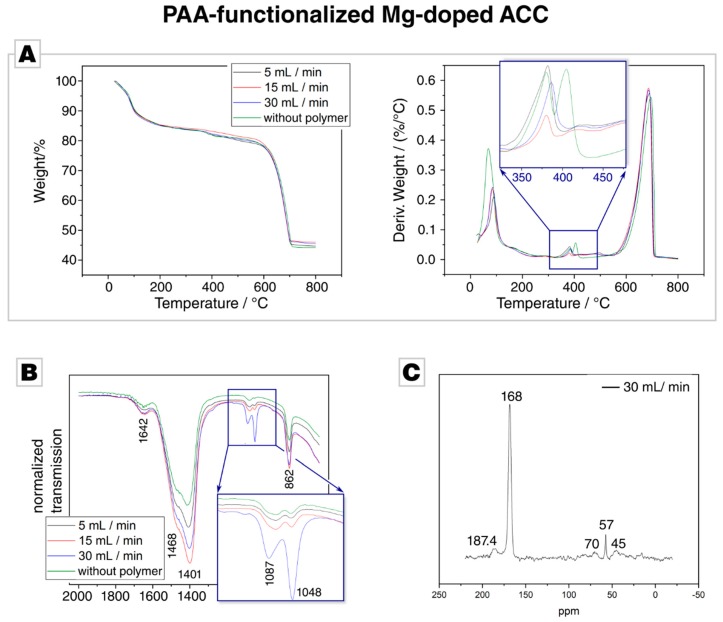
(**A**) Thermal gravimetric analysis of the PAA experiments and the Mg-doped ACC control sample. The different PAA-functionalized samples show no significant variation. (**B**) Fourier transform infrared spectrum of the PAA-functionalized and the control samples; the complete spectrum is provided in Figure S3B in the Supplementary Materials. (**C**) CP-MAS ^13^C–SS-NMR of PAA-functionalized Mg-doped ACC synthesized at 30 mL/min. The signals at 39 to 48 ppm can be attributed to overlapping signals of the CH and CH_2_ moieties in the vinyl backbone of PAA; the signal at 185 ppm arises from carboxylic moieties of the PAA polymer. Other signals (e.g., at 57 ppm) arise from impurities, such as residual ethanol, or are uninformative to the signal/noise ratio.

TGA analyses of the PAA-functionalized precipitates show, below 100 °C, a distinct weight loss of about 10% occurs, which we attribute to the loss of water (Figure 4A). PAA degradation occurs over a wide temperature range from 250 °C to 500 °C [73]. In this regime, also the polymer-free control sample shows a distinct weight loss which we link to a loss of chemically bound water. The kink, which is caused by the decomposition of MgCO_3_, is present in all samples. Remarkably, this decomposition occurs—like in the case of PSS—in two steps when no polymer is present but in a single step if PAA is present. We have currently no explanation for this behaviour, but it may indicate a different structural organisation of the PAA and PSS samples, that is, two different Mg-related chemical environments both present in the polymer-free and PSS-containing ACC but only one present in PAA-containing ACC. After 400 °C, the derivative weight loss is distinctly higher in the case of PAA samples than for the control samples. This might arise from decomposing PAA, whose thermolysis occurs in the temperature range of 250 °C to 500 °C [73]. Similar to the case of PSS-functionalized ACC particles, TGA evidenced the presence of Mg incorporated in the PAA-functionalized ACC, but the presence of PAA remained unsettled.

ATR-FTIR analysis was conducted, since the question whether PAA is present in these precipitates remained unanswered by TGA. All spectra showed the relevant band for ACC but gave no direct evidence for the presence of PAA in the precipitate (Figure 4B, the spectrum of the employed PAA is given in Figure S2B of the Supplementary Materials). The band at 1048 cm^−1^, which is also present in control and PSS samples, is remarkably pronounced in the presence of PAA and further increases with higher flow rates (see inset in Figure 4B); a behaviour which was not observed in the case of PSS. As the band is also present in the control sample, it becomes clear that this band is not connected with an incorporated polymer. Instead, this band probably arises from the hydration of the ACC; Jensen et al. described a shoulder at 1050 cm^−1^ in ACC spectra, which decreases with dehydration [66]. This assumption is backed by the band intensity at 3400 cm^−1,^ which stems from OH stretching vibrations [74]. With an increasing flow rate, the intensity of this band increases in case of the PAA samples (see Figure S3B in the Supplementary Materials). As PAA is known to be a strong water sorbent [75], the behaviour of this band indicates that polyacrylates are indeed incorporated into the precipitate and lead to slightly more occluded water; an assumption which is backed by a slightly higher amount of water loss in the TGA measurements of PAA samples in comparison to PSS samples. In summary, the ATR-IR analysis could only give indirect evidence of PAA incorporation into the Mg-doped ACC, but shows that the hydration rate of the precipitate is affected when PAA is used as a polymeric additive.

CP-MAS ^13^C–SS-NMR spectra showed a distinct dependence on the flow rate for the PAA-samples, indicating a sensitivity of the PAA incorporation rate on the flow rate. At a low flow rates, the NMR spectra show only one dominant, strong resonance which arises from carbonate, but no PAA signals could be detected; overall, the spectra were fully consistent with those acquired in the PSS case and thus did not indicate the incorporated polymer. At higher flow rates, new resonances appear. The ^13^C CP spectrum of the sample synthesised at a flow rate of 30 mL/min was recorded with a recycle delay of 3 s and showed the expected signal of amorphous carbonate at ca. 168.4 ppm [76], with a full width at half height (FWHH) of about 375 Hz. This chemical shift nicely correlates with the shift of crystalline calcium carbonate at 168.2 ppm [72]. The signal is, however, significantly broadened compared to synthetic and geological crystalline calcium carbonate [32,36]. Thus, the enlarged FWHH, being a measure for the local order in a system [32,36], confirms the amorphous nature of the sample. Additionally, broad ^13^C resonances at 187.4 ppm and 44.9 ppm for the side chain COOH group and the backbone alkyl CH and CH_2_ groups were also detected in the CP spectrum [32]. Weak signals at ca. 57 and 70 ppm arose from trace contaminations: Residual ethanol was used to remove adhered water during the drying process [70].

## 4. Conclusions and Outlook

In this work, we demonstrated that it is possible to reliably generate Mg-doped amorphous calcium carbonate by a simplistic flow-chemistry approach by exploiting commercially available microfluidic chips connected in series. The consecutive layout of the flow-chemistry experiments thus allows for subsequent functionalization steps of the nanoscaled precipitate: Here, with a thin polymeric corona made from PAA or PSS. We demonstrated further that the flow rate affects the composition of the amorphous co-precipitate: Here, the Mg/Ca ratio. The polymer can also have a flow rate dependent effect on the composition, as demonstrated by the case of PAA in which the hydration rate of the ACC is slightly affected. However, this phenomenon is polymer-dependent: PSS does not show such a flow rate dependent behaviour. In the future, we will expand on this, aiming at subsequent self-organisation steps to generate architectured and functionally or chemically graded materials. The combination of a well-controllable flow-chemistry setup with subsequent nanoparticle self-assembly may be thus a new and bio-inspired route to produce cost-effective gradient materials that may be used in a variety of different fields, such as in the generation of lightweight construction elements [77] or the development of tuneable bioactive coatings [17].

## Supplementary Materials

The following are available online at https://www.mdpi.com/1996-1944/12/11/1818/s1, Figure S1: X-ray diffractograms of Mg-doped ACC generated in the flow-chemistry setup in presence of PSS (A) and PAA (B), Figure S2: ATR-FTIR spectra of the pure polymers PSS (A) and PAA (B), Figure S3: ATR-FTIR spectra of (A) PSS-functionalized and (B) PAA-functionalized Mg-doped ACC, prepared at varying flow rates.

## Appendix A

It should be noted that, according to the ISO Technical Specification 80004, particles are not nanoparticles by definition if at least one of their external dimensions exceeds the length interval approximately from 1 nm to 100 nm. For the sake of manuscript conciseness and readability we omit this discrimination.
