# Supplementary material for: Polymer-Functionalised Nanograins of Mg-Doped Amorphous Calcium Carbonate via a Flow-Chemistry Approach
Source: Materials (Basel). 2019 Jun 4;12(11):1818. doi: 10.3390/ma12111818 (PMC6601056; doi:10.3390/ma12111818)
Supplement: Supplementary file 1 [file materials-12-01818-s001.pdf]

Supplemental Information for

Polymer-functionalized nanograins of Mg-doped amorphous calcium carbonate via a flow-chemistry approach

Benedikt Demmert[1,4], Frank Schinzel[1], Martina Schüßler[1], Mihail Mondeshki[2], Joachim Kaschta[3], Dirk W. Schubert[3], Dorrit E. Jacob[4], and Stephan E. Wolf[1,5,*]

1 Department of Materials Science and Engineering (WW), Institute of Glass and Ceramics (WW3), Friedrich-Alexander University Erlangen-Nuremberg (FAU), Martensstrasse 5, D-91058 Erlangen, Germany
2 Institute for Inorganic and Analytical Chemistry, Johannes Gutenberg-University, Duesbergweg 10-14, 55128 Mainz, Germany
3 Department of Materials Science and Engineering (WW), Institute of Polymer Materials (WW5), Friedrich-Alexander University Erlangen-Nuremberg (FAU), Martensstrasse 5, D-91058 Erlangen, Germany
4 Department of Earth and Planetary Sciences, Macquarie University, Sydney, 2109 NSW, Australia
5 Interdisciplinary Center for Functional Particle Systems (FPS), Friedrich-Alexander University Erlangen-Nuremberg, 91058 Erlangen, Germany
* Correspondence: stephan.e.wolf@fau.de

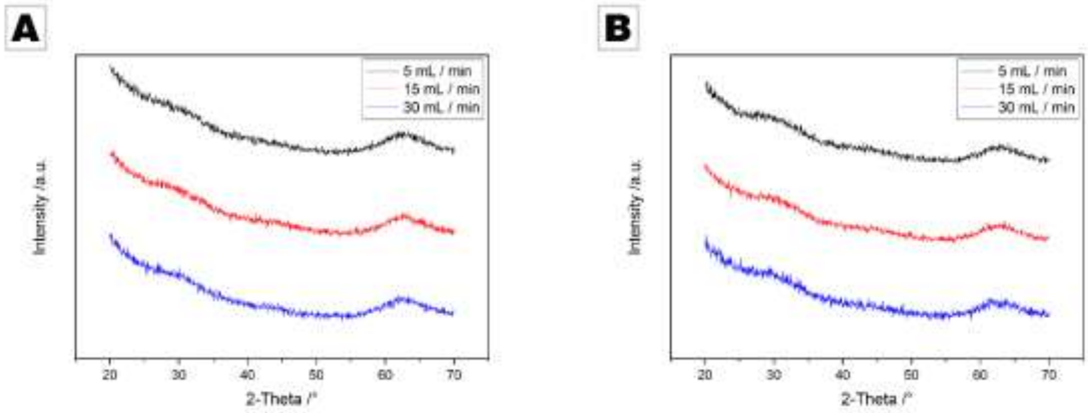

Figure S1. X-ray diffractograms of Mg-doped ACC generated in the flow-chemistry setup in the presence of PSS **(A)** and PAA **(B)**.

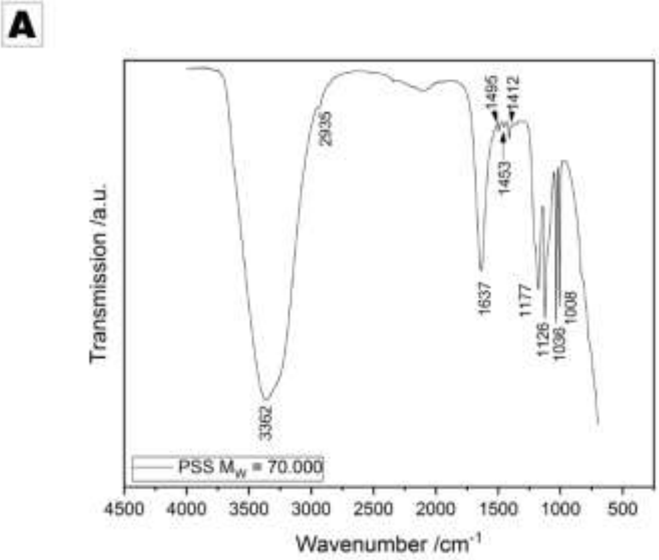

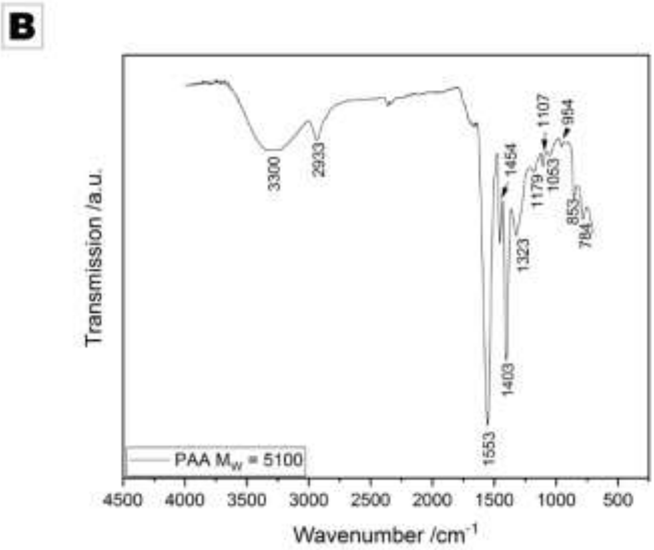

Figure S2. ATR-FTIR spectra of the pure polymers PSS **(A)** and PAA **(B)**.

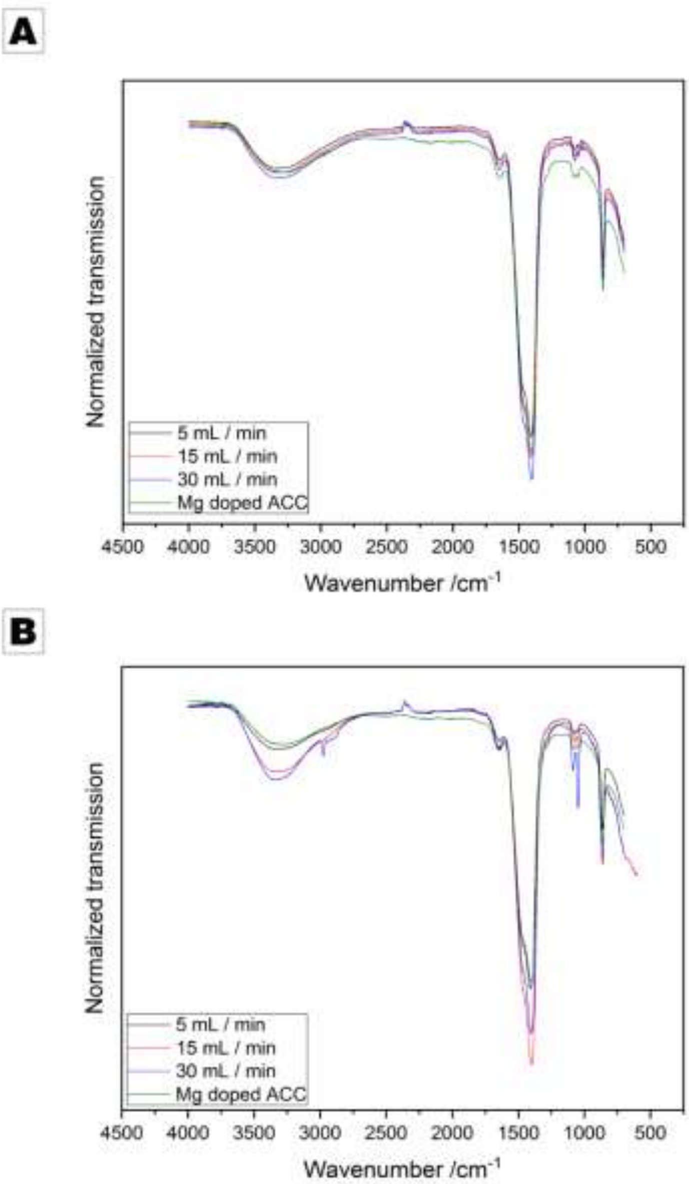

Figure S3. ATR-FTIR spectra of **(A)** PSS-functionalized and **(B)** PAA-functionalized Mg-doped ACC, prepared at varying flow rates. In the range from 3000 to 2700 cm^{-1} only bands arising from solvents are detected, i.e., water or ethanol. In the case of PAA, the water bands at 1048 cm^{-1} and 3400 cm^{-1} increase with increasing flow rate.
